# The roles of chemokines following intracerebral hemorrhage in animal models and humans

**DOI:** 10.3389/fnmol.2022.1091498

**Published:** 2023-01-10

**Authors:** Jinjin Wang, Liheng Bian, Yang Du, Dandan Wang, Ruixuan Jiang, Jingjing Lu, Xingquan Zhao

**Affiliations:** ^1^Department of Neurology, Beijing Tiantan Hospital, Capital Medical University, Beijing, China; ^2^China National Clinical Research Center for Neurological Diseases, Beijing, China; ^3^Research Unit of Artificial Intelligence in Cerebrovascular Disease, Chinese Academy of Medical Sciences, Beijing, China; ^4^Beijing Institute of Brain Disorders, Collaborative Innovation Center for Brain Disorders, Capital Medical University, Beijing, China

**Keywords:** chemokines, receptors, roles, intracerebral hemorrhage, preclinical study, clinical study

## Abstract

Intracerebral hemorrhage (ICH) is one common yet devastating stroke subtype, imposing considerable burdens on families and society. Current guidelines are limited to symptomatic treatments after ICH, and the death rate remains significant in the acute stage. Thus, it is crucial to promote research to develop new targets on brain injury after ICH. In response to hematoma formation, amounts of chemokines are released in the brain, triggering the infiltration of resident immune cells in the brain and the chemotaxis of peripheral immune cells *via* the broken blood–brain barrier. During the past decades, mounting studies have focused on the roles of chemokines and their receptors in ICH injury. This review summarizes the latest advances in the study of chemokine functions in the ICH. First, we provide an overview of ICH epidemiology and underlying injury mechanisms in the pathogenesis of ICH. Second, we introduce the biology of chemokines and their receptors in brief. Third, we outline the roles of chemokines in ICH according to subgroups, including CCL2, CCL3, CCL5, CCL12, CCL17, CXCL8, CXCL12, and CX3CL1. Finally, we summarize current drug usage targeting chemokines in ICH and other cardio-cerebrovascular diseases. This review discusses the expressions of these chemokines and receptors under normal or hemorrhagic conditions and cell-specific sources. Above all, we highlight the related data of these chemokines in the progression and outcomes of the ICH disease in preclinical and clinical studies and point to therapeutic opportunities targeting chemokines productions and interactions in treating ICH, such as accelerating hematoma absorption and alleviating brain edema.

## 1. Introduction

### 1.1. Epidemiology of intracerebral hemorrhage

Intracerebral hemorrhage (ICH), or hemorrhagic stroke, is one common yet disproportionately deadly stroke subtype, with a significant death rate and morbidity among survivors (Collaborators, 2021; O’Carroll et al., 2021). Spontaneous ICH is defined as bleeding into the brain parenchyma due to the rupture of cerebral blood vessels resulting from hypertension or cerebral amyloid angiopathy (CAA). Given the data from the Global Burden of Disease Study 2019, ~3.41 million patients were first diagnosed with hemorrhagic strokes worldwide each year, whereas the absolute number of deaths was 2.89 million caused by acute compression and related complications after ICH (Collaborators, 2021). Nowadays, there is a total of 20.66 million patients with current ICH worldwide, placing substantial burdens on families and society for post-stroke care and rehabilitation.

During the past decades, the rapid advance has been achieved in new therapeutic strategies, thereby contributing to prognosis improvements, such as acute blood pressure management and minimally invasive surgery for ICH (Xue and Yong, 2020; Greenberg et al., 2022). However, the death rate remains notably high at the ultra-early stage of ICH. Thus, it is crucial to clarify the etiology, pathogenesis, and underlying injury mechanisms for establishing reliable prognostic biomarkers in predicting prognosis, prompting specific therapies in managing ICH, and reducing the devastating effects of ICH eventually.

### 1.2. Injury mechanisms of intracerebral hemorrhage

Intracerebral hemorrhage is a complex pathophysiology marked by primary and secondary injury. Initial bleeding leads to hematoma formation, mass effects, and further high intracranial pressure, contributing to compressed brain tissue, broken blood flow, and even cerebral hernia. Secondary injury results from a cascade of events initiated by the hematoma and corresponding body responses, such as hemostasis, inflammation, and erythrocyte lysis (Keep et al., 2012).

#### 1.2.1. Inflammatory and immune cells

Mounting evidence has demonstrated that ICH is a systemic disease affecting more than the brain (Saand et al., 2019). Once ICH occurs, systemic inflammation and immune responses are rapidly activated and exert an essential role in stroke-related injury and recovery, characterized by resident immune cells in the central nervous system (CNS) and infiltrated cells in the peripheric immune system. Microglia is recognized as the first immune cell activated by brain injury within a few minutes of ICH onset and acts as the primary source of inflammatory factors at the early stage (Wang, 2010). The role of microglia following ICH is complex (Liu et al., 2021). Contrarily, microglia also play a neuroprotective effect on anti-inflammatory and neural repair actions by releasing anti-inflammatory cytokines, extracellular matrix proteins, and other substances (Zlokovic, 2008). Astrocyte intertwines with blood vessels and synapses, affecting neuronal function and blood flow after ICH (Scimemi, 2018). A wide variety of molecules are secreted by astrocytes, including proinflammatory cytokines (IL-6 and IL-1β), anti-inflammatory cytokines (IL-10), and chemokines (CCL2, CXCL1, CXCL10, and CXCL12), through which controlling microglia differentiation and macrophage activation (Mantovani et al., 2004; McKimmie and Graham, 2010; Allaman et al., 2011; Nash et al., 2011; Lan et al., 2017).

Human data have consistently suggested that bleeding triggers a rapid increase of peripheral leukocyte counts that mainly infiltrates the brain parenchyma through the damaged blood–brain barrier (BBB), dependent on cytokines and chemokines. Previous studies reported that neutrophil was the first infiltrating immune cell detected in the perihematomal region at 5–8 h and remained at a high level at 12 days of ICH onset (Mackenzie and Clayton, 1999; Shtaya et al., 2019). However, Li and his colleagues recently identified the swift arrival of natural killer (NK) cells to the perihematomal regions within 12 h of ICH onset, even earlier than other mobilized neutrophils and T cells (Li et al., 2020). Lymphocytes were mainly observed in the perihematomal brain with lower concentrations on days 1–3, with moderate accumulation later (Mackenzie and Clayton, 1999; Shtaya et al., 2019). For monocyte/macrophage, it was found to migrate into the injury site within 12 h and peaked at days 3–5 post-ICH (Hammond et al., 2012; Bonsack et al., 2016). Increased leukocyte counts and related indexes are associated with disease severity, in-hospital death, and poor outcomes in hemorrhagic strokes (Du et al., 2022; Wang et al., 2022).

#### 1.2.2. Inflammatory and immune factors

In the inner responses of post-stroke, inflammatory and immune mediators play a major role in the acute injury and ensuing recovery, mainly including cytokines and chemokines. Cytokines are small proteins secreted by various cell types, especially activated lymphocytes and macrophages. It regulates the balance between cellular and humoral immunity (Zhu et al., 2019). Like cytokines, chemokines also belong to a family of small proteins constitutively produced by leukocytes and tissue cells or induced after events. However, chemokines are much smaller than cytokines and exert their effects *via* heptahelical G-protein coupled receptors (GPCRs), typical for leukocyte attractants (Baggiolini, 2001). Chemokines contain roughly 70–130 amino acids (Baggiolini et al., 1997). The function of chemokines in ischemic stroke has been well documented, but their role in hemorrhagic stroke has not been completely understood. Specially, we examined potential cellular and molecular mediators from animal and human data in ICH progression and prognosis. This review summarizes the emerging evidence describing the chemokines and their receptors following ICH in preclinical and clinical studies.

## 2. Biology of chemokines and their receptors

Chemokines were first identified in the late 1980s. In the early 1990s, IL-8 (CXCL8) was reported to be involved in recruiting neutrophils in acute inflammation models (Matsushima, 2000; Zlotnik and Yoshie, 2012). According to the positions of critical cysteine residues, chemokines are subdivided into C, CC, CXC, and CX3C subgroups. CXC, CC, and CX3C chemokines have four conserved cysteines, while C chemokines have only two cysteines. CXC and CX3C chemokines are distinguished by the presence of one (CXC) or three (CX3C) amino acids between the first and second cysteines, but the first two cysteines of CC chemokines are adjacent (Bajetto et al., 2001). Chemokines activate several different signal transduction pathways *via* binding with their specific or shared GPCRs. Most receptors are also grouped into four subfamilies according to their major chemokine ligands, including the CXCR subfamily, CCR subfamily, XCR subfamily, and CX3CR subfamily, except for several atypical receptors (Zlotnik and Yoshie, 2012).

The chemokine family is classified as inflammatory, homeostatic, and dual-functional factors according to their chemotaxis to leukocytes or/and subsets of lymphocytes or dendritic cells (Zlotnik and Yoshie, 2012). Chemokines participate in the inflammation, immune responses, and immune system establishment. In addition, they regulate angiogenesis in inflammation, neoplasia, reproductive systems, hematopoietic systems, and organ development (Matsushima, 2000).

All microglial cells, astrocytes, and neurons can secrete chemokines. Under physiological conditions, homeostatic chemokines can originate from the vasculature of blood-brain barrier (BBB) to participate in the development of CNS, regulating the entry of leukocytes for immunosurveillance, such as CXCL12, CCL19, CCL20, and CCL21 (Chen K. et al., 2018). Unlike homeostatic chemokines, inflammatory chemokines are expressed by numerous cell types at almost any tissue location and remain low in the CNS. However, their expressions increase significantly on circulating leukocytes and other cell types activated by acute brain injury (Yao and Tsirka, 2012b; Le Thuc et al., 2015), allowing for recruiting and positioning immune cells in the damaged tissues (Yao and Tsirka, 2012b; Le Thuc et al., 2015; Kothur et al., 2016; Chen C. et al., 2018; Trettel et al., 2020). In the procedure of ICH, specific chemokines and their receptors play an essential part in secondary injury and neurological recovery (Table 1). Next, we will discuss their roles following ICH according to their subfamilies and summarize the data in preclinical and clinical studies (Tables 2, 3).

**Table 1:** Summary of chemokines and their receptor.

Subfamily	Main functions	Receptor	Cell expression	Chemokine ligand(s)	Reference
C C chemokine	Regulate the infiltration of monocytes, basophils, eosinophils, and T lymphocytes but have little effect on neutrophils.	CCR1	Monocytes, macrophages, neutrophils, T cells, basophils, microglia, neurons, astrocytes	CCL3, CCL5, CCL7, CCL8, CCL13, CCL14, CCL15, CCL16, CCL23	White et al. (2013)
		CCR2	Monocytes/macrophages, endothelial cells, leukocytes, smooth muscle cells, basophils, DC, natural killer cells, activated T lymphocytes, microglia, astrocyte, neurons, brain microvascular endothelial cells	CCL2, CCL7, CCL8, CCL12, CCL13, CCL16	Chu et al. (2014) and França et al. (2017)
		CCR3	Eosinophil, basophils, mast cells, vascular endothelial cells, astrocytes, microglia, oligodendrocytes	CCL5, CCL11, CCL13, CCL24	Pease and Horuk (2014) and Huber et al. (2018)
		CCR4	T cells, astrocytes, microglia, neurons	CCL17, CCL22	Scheu et al. (2017)
		CCR5	Resting memory/effector T-lymphocytes, monocytes, macrophages, immature dendritic cells, astrocytes, microglia, neurons	CCL3, CCL5, CCL8	Blanpain et al. (2002)
		CCR6	Treg cells, Th17 cells	CCL20	Meitei et al. (2021)
		CCR7	B cells, mature DC, T cells, neurons	CCL19, CCL21	Liu et al. (2007) and Förster et al. (2008)
		CCR8	Th2 cells, Treg cells, Tconv cells, NK cells, thymocytes, endothelial cells, neurons	CCL1, CCL8, CCL18	Liu et al. (2007) and Moser (2022)
		CCR9	DC, neutrophils, lymphocytes, monocytes, macrophages, and vascular endothelial cells, neurons	CCL25	Liu et al. (2007) and Wu et al. (2021)
		CCR10	T cells, Langerhans cells, melanocytes, endothelial cells, microvascular endothelial cells, fibroblasts, neurons	CCL27, CCL28	Liu et al. (2007) and Xiong et al. (2012)
XC chemokine	Regulate DC-mediated cytotoxic immune response and the thymic establishment of self-tolerance and the generation of regulatory T cells	XCR1	DC subpopulation, CD8^+^ cells, NK cells, B cells, CD3^+^ T cells, CD4^+^ T cells, neutrophils	XCL1, XCL2	Lei and Takahama (2012)
CXC chemokine	Attract neutrophils, lymphocytes, and monocytes	CXCR1	Neutrophils, CD8 T cells, monocytes, NK cells, mast cells, fibroblasts, endothelial cells, SMCs, astrocytes, microglia, neurons, oligodendrocytes	CXCL1, CXCL2, CXCL3, CXCL4, CXCL6, CXCL8	Mamik and Ghorpade (2016) and Dhayni et al. (2022)
		CXCR2	Neutrophils, CD8 T cells, monocytes, NK cells, mast cells, fibroblasts, endothelial cells, SMCs, astrocytes, microglia, neurons, oligodendrocytes	CXCL1, CXCL2, CXCL3, CXCL4, CXCL5, CXCL6, CXCL7, CXCL8	Semple et al. (2010), Mamik and Ghorpade (2016) and Dhayni et al. (2022)
		CXCR4	Lymphocytes, endothelial, epithelial and hematopoietic stem cells, stromal fibroblasts, neurons, microglia, astrocytes, oligodendrocytes	CXCL12	Jiang et al. (2013) and Pozzobon et al. (2016)
		CXCR5	Mature B cells, subpopulations of CD4^+^ T cells, DCs	CXCL13	Pan et al. (2022)
CX3C chemokine	Induce the chemotaxis of monocytes and cytotoxic T cells.	CX3CR1	Monocytes, NK cells, T cells, smooth muscle cells microglia, neurons, astrocytes	CX3CL1	Subbarayan et al. (2022)

Th, T helper; Treg cells, regulatory T cells; DC, dendritic cells; CD, cluster of differentiation; NK, natural kill; SMC, smooth muscle cells.

**Table 2:** Summary of animal studies investigating the role of chemokines in intracerebral hemorrhage.

Chemokine	Receptor	Species	ICH Model	Methods of inhibition/activation	Time	Effect of chemokines/receptors	References
CCL2	CCR2	C57BL/6 mice	Collagenase	Genetic deletion	1 day, 3 days, 7 days, 14 days	CCL2/CCR2 deficiency might decrease hematoma size at early time points but delay the recovery.	Yao and Tsirka (2012a)
CCL2	CCR2	SD rat	Collagenase	Pharmacological intervention (CCR2 inhibitor propagermanium, oral gavage, three times a day, 25 mg/kg/day for 1 day or 3 days)	1 day, 3 days	Propagermanium maintain BBB integrity, reduce brain edema, and improve neurobehavioral functions.	Guo et al. (2020)
CCL2	CCR2	SD rat	Collagenase	Pharmacological intervention (S1PR3 antagonist CAY10444, I.P., 0.5 mg/kg/day for 1 day or 3 days)	1 day, 3 days	S1PR3 inhibition exerts a neuroprotective effect *via* the S1P-CCL2-p-p38 MAPK pathway.	Xu et al. (2021)
CCL2	CCR2	C57BL/6 mice	Collagenase	Genetic deletion; Pharmacological intervention (CCR2 antibody MC-21, I.P., 20 μg before ICH and again 24 h later)	1 day, 3 days, 7 days	CCR2 deletion and MC-21 decreased inflammatory monocyte recruitment and are protected from early motor deficits	Hammond et al. (2014)
CCL2	/	mouse	Autologous blood	Gene silencing by shRNA	48 h	MCP-1 shRNA inhibited inflammation response and improved neurological injury	Yang et al. (2016)
CCL5	CCR5	CD1 mice	Autologous blood	Pharmacological intervention (CCR5 antagonist Maraviroc, intranasally, 50 μg/kg or 150 μg/kg or 450 μg/kg per day for 3 days)	3 h, 6 h, 12 h, 24 h, 72 h	MVC improved neurobehavioral deficits and decreased neuronal pyroptosis in ipsilateral brain tissues, partially through the CCR5/PKA/CREB/NLRP1 signaling pathway	Yan et al. (2021)
CCL5	CCR1	CD1 mice	Autologous blood	Pharmacological intervention (CCR1 inhibitor Met-RANTES, intranasally, 0.15 μg/kg or 0.5 μg/kg or 1.5 μg/kg at 1 h post-ICH)	3 h, 6 h, 12 h, 24 h, 72 h	CCR1 inhibition with Met-RANTES attenuated neuroinflammation, thereby reducing brain edema and improving neurobehavioral functions	Yan et al. (2020)
CCL5	CCR1	CD1 mice	Autologous blood	Pharmacological intervention (CCR1 antagonist Met-RANTES, intranasally, 0.15 μg/kg or 0.5 μg/kg or 1.5 μg/kg at 1 h post-ICH)	1 h, 24 h, 72 h, 7 days, 14 days, 21 days, 25 days	Met-R treatment attenuated blood–brain barrier permeability and ameliorated neurobehavioral deficits through inhibiting CCR1/SRC/Rac1 signaling pathway in mice.	Yan et al. (2022)
CCL12	/	C57BL/6 mice	Autologous blood	Genetic deletion. Pharmacological intervention (CCL12 antibody, I.P.)	1 day, 3 days, 5 days, 7 days	CCL12 deletion attenuated ICH damage in the brain	Huang et al. (2020)
CCL17	CCR4	CD1 mice	Autologous blood	Pharmacological intervention (CCR4 activator rCCL17, intranasally, 10 μg/kg, 30 μg/kg, 90 μg/kg, at 1 h following ICH)	6 h, 12 h, 24 h, 72 h, 5 days, 7 days, 14 days, 21 days, 22–27 days	CCR4 activation with rCCL17 promoted hematoma resolution by increasing CD163 expression and CCR4/ERK/Nrf2 pathway, thereby reducing brain edema and improving neurological function	Deng et al. (2020)
CCL17	CCR4	CD1 mice	Autologous blood	Pharmacological intervention (CCR4 activator, rCCL17, 10 μg/kg, 30 μg/kg, 90 μg/kg, intranasally at 1 h following ICH)	6 h, 12 h, 24 h, 72 h, 5 days, 7 days, 14 days, 21 days, 22–27 days	rCCL17-dependent CCR4 activation ameliorated neurological deficits, reduced brain edema, and ameliorated neuroinflammation and neuronal apoptosis, at least in part, through the PI3K/AKT/Foxo1 signaling pathway after ICH	Deng et al. (2021)
CXCL2	CXCR1/2	C57BL/6J mice	Collagenase	Pharmacological intervention (CXCR1/2 antagonist, reparixin)	0, 3 h, 6 h, 12 h, 24 h	CXCR1/2 antagonist reparixin ameliorated neurological deficits	Matsushita et al. (2014)
/	CXCR4	SD rats, C57BL/6 mice	Collagenase	Pharmacological intervention (CXCR4 agonist CX807, I.P., 3 mg/kg/day for 3 days)	30 min, 60 min, 90 min, 3 days, 4 days	CX807 is neuroprotective and anti-inflammatory against ICH	Yu et al. (2020)
CXCL12	CXCR4	SD rats	Collagenase	Pharmacological intervention (CXCL12, I.V., 10 μL every other day; CXCR4 agonist, AMD3100, S.C., 120 μg/kg, twice daily)	24 h, 14 days	CXCL12 stimulates EPCs to induce angiogenesis though the CXCR4 pathway after ICH	Li et al. (2015)
CX3CL1	CX3CR1	C57BL/6 mice	Collagenase	Pharmacological intervention (CX3CL1 and a CX3CR1 inhibitor AZD8797, lateral ventricular injection)	6 h, 12 h, 24 h, 3 days, 7 days	CX3CL1 significantly decreased the hematoma size and Hb content and improved neurological deficits	You et al. (2022)
CX3CL1	CX3CR1	C57BL/6 mice	Collagenase	Genetic overexpression	6 h, 12 h, 1 day, 2 days, 3 days, 5 days, 7 days	The overexpression of CX3CR1 increased the migration ability of adipose derived stem cells, reduced cell death and improved sensory and motor functions	Li G. et al. (2019)

BBB, blood–brain barrier; I.P., intraperitoneal injection; I.V., intravenous injection; S.C., subcutaneous injection; EPCs, endothelial progenitor cells.

**Table 3:** Summary of human studies investigating the role of chemokines in intracerebral hemorrhage.

Chemokine/receptor	Study	Population	Sample source	Time	Effects of chemokines/receptors	References
CCL2, CXCL10	prospective cohort	115 ICH patients	Serum	6 h, 24 h, 72 h	Patients with elevated CCL2 had worse mRS score at day 90. Elevation in CXCL10 was independently associated with worse 90-d mRS score	Landreneau et al. (2018)
CCL2	prospective, multi-center	28 ICH/IVH patients	CSF, serum	1–10 days	Significant correlation was found between CCL2 level and IVH volume at 3–8 days and ICH at day 1–2. Significant correlations were found with PHE volume for IL-6, IL-10 and CCL2 at day 1–2	Ziai et al. (2021)
CCL2	prospective cohort	85 ICH patients	Serum	24 h	Serum CCL2 levels are independently associated with functional outcomes	Hammond et al. (2014)
CCL5	prospective cohort	15 ICH patients, 10 controls	Serum	1–3 days, 7 days, 14 days, 30 days	No significant association was found	Li et al. (2012)
CCL23	prospective	94 ICH patients, 47 controls	Serum	Within 24 h	Serum CCL23 levels was highly related to ICH severity and inflammatory response; CCL23 ≥ 62.95 pg./mL served as an independent predictor of 6-month unfavorable outcome and death	Lin et al. (2022)
CXCL8	/	4 deceased ICH patients, 24 ICH patients, 20 controls	Brain samples, serum	Within 24 h	CXCL8 elevated in blood samples of ICH patients	Rosell et al. (2011)
CXCL12	cohort	105 ICH patients, 105 healthy controls	Serum	Within 24 h	CXCL12 concentrations had positive correlation with NIHSS scores and hematoma volume. Serum CXCL12 was independently associated with the mortality, overall survival, and unfavorable outcome	Shen et al. (2017)
CX3CL1/CX3CR1	prospective cohort	30 ICH patients	Serum	Within 7 days	Serum CX3CL1 concentration was relative to smaller hematoma volume and better outcome	You et al. (2022)
CX3CL1/CX3CR1	/	15 TBI/ICH patients, 5 patients with unruptured intracranial aneurysms	Brain tissue	/	Higher CX3CR1 expression in neurons was associated with better clinical conditions of patients at admission	Gaetani et al. (2013)

ICH, intracerebral hemorrhage; mRS, modified Rankin Scale; IVH, intraventricular hemorrhage; CSF, cerebrospinal fluid; TBI, traumatic brain injury.

## 3. Chemokines and their receptors in intracerebral hemorrhage

### 3.1. CC chemokine family

The members of the CC chemokine family usually relate to the infiltration of monocytes, basophils, eosinophils, and T lymphocytes but have little effect on neutrophils (Bajetto et al., 2001). The most studied CC chemokines associated with ICH were CCL2, CCL3, CCL4, CCL5, CCL17, and CCL20.

#### 3.1.1. CCL2

CCL2, also known as monocyte chemoattractant protein-1 (MCP-1), is the first human CC chemokine on chromosome 17 (chr.17, q11.2). Human CCL2 consists of 76 amino acids, and the molecular weight is 13 kDa (Van Coillie et al., 1999). Many types of cells can produce CCL2, such as monocytes/macrophages, endothelial cells, fibroblasts, epithelial cells, smooth muscle cells, and mesangial cells. In the CNS, CCL2 is predominantly secreted by astrocytes, resident microglial cells, neurons, and endothelial cells. The monocyte/macrophage has been found as the primary source (Deshmane et al., 2009). CCL2 exerts effects by bonding its cognate receptor CCR2, although it can bind to CCR4 expressed on Th2 lymphocytes (Zhang et al., 2006). Unlike CCL2, CCR2 is relatively restricted to certain types of cells according to the forms of CCR2, including CCR2A and CCR2B. In the peripheral system, CCR2A is the major isoform expressed by mononuclear cells and vascular smooth muscle cells, whereas CCR2B is predominantly expressed by monocytes and activated NK cells (Deshmane et al., 2009; Chu et al., 2014). In the CNS, CCR2 expresses on the surfaces of microglia, astrocyte, neurons, and brain microvascular endothelial cells (Semple et al., 2010).

CCL2/CCR2 axis mainly induces the egression and chemotaxis of monocyte/macrophage (Bajetto et al., 2001), shaping macrophage polarization to participate in the inflammation process (Sierra-Filardi et al., 2014). In addition, the network is vital in regulating neutrophils, T lymphocytes, and NK cells (van Helden et al., 2012; Vasanthakumar et al., 2020; Shibuya et al., 2022). The production of CCL2 is constitutive in specific cells, but its expression is upregulated by proinflammatory cytokines such as IL-1β and tumor necrosis factor (TNF)-α, growth factors, reactive oxygen species (ROS), and oxidized low-density lipoproteins (oxLDLs; Barlic and Murphy, 2007; Hinojosa et al., 2011; Gruber et al., 2015; Bianconi et al., 2018; Akhter et al., 2021). The upregulated CCL2 recruits immune cells into inflamed tissues by binding to CCR2. Recent studies have also reported the role of CCL2/CCR2 in promoting angiogenesis and regeneration (Pan et al., 2020). In cerebral ischemic stroke, this axis is involved in neuroinflammation and contributes to brain injury in the acute stage (Cisbani et al., 2018), but also promotes the migration of neuroblasts and neurological recovery (Pedragosa et al., 2020; Geng et al., 2022).

CCL2 has been studied widely in ICH. The functional network map indicates that CCL2 is a key molecule in the pathogenesis of the innate immune response and regulation of the immune effector process after ICH (Xu et al., 2021). CCL2 is produced by astrocytes, microvascular endothelial cells, microglia, and neurons, whereas CCR2 is detected on the surface of astrocytes and brain microvascular endothelial cells (BMECs; Guo et al., 2020). In the autologous blood models of the mouse, CCL2 was detected elevated at day 1, continuously higher up to 3 days, and decreased at 7 days after ICH. Another study showed an elevation of CCL2 at 12 h after experimental ICH induced by collagenase (Hammond et al., 2014; Huang et al., 2022). CCL2/CCR2 system mediates inflammation after ICH. CCR2^−/−^ mice exhibited decreased recruited monocytes and fewer motor deficits in the early ICH (Hammond et al., 2014). CCL2/CCR2 knockout could also inhibit the proliferation and cytotoxicity of microglial cells, reduce infiltration of monocytes in the brain, ameliorate neurological deficits, and improve brain edema (Hammond et al., 2014; Yang et al., 2016). Receptor-interacting protein kinase 3 (RIPK3), a key kinase in the necroptosis pathway, may interact with CCL2 to modulate inflammatory responses and RIPK3-dependent necroptosis (Huang et al., 2022). S1PR3 modulator CAY10444 also could alleviate early inflammation and exert neuroprotective effects *via* the S1P-CCL2-p-p38 MAPK pathway (Xu et al., 2021). The expression of CCL2 has also been found to contribute to BBB disruption *via* the p38 MAPK signaling pathway following ICH (Guo et al., 2020). Consequently, the administration of CCR2 inhibitor propagermanium (PG) effectively maintained the BBB integrity, reduced brain edema, and improved neurobehavioral functions. Moreover, the CCL2-CCR2 signaling pathway affects the progression and resolution of the hematoma. At early times, CCL2/CCR2 deficiency might decrease hematoma size but delay long-term recovery (Yao and Tsirka, 2012a). This may be associated with inflammation being a double-edged sword, with beneficial or detrimental effects depending on the timing and environment.

In human studies, a small-sample study showed that cerebrospinal fluid (CSF) CCL2 peaked early on days 1–2 and then decreased in patients with spontaneous intracerebral ventricular hemorrhage (IVH; Ziai et al., 2021). The serum concentration of CCL2 was found to increase on days 1–3 after onset and then dropped slightly on days 7–14 but elevated after 14 days (Li et al., 2012). CCL2 has been thought to be an indicator of early ICH severity. The analysis of human PHE tissue showed an elevated level of CCL2. A positive relationship was observed between elevated CCL2 expression and PHE volume (Guo et al., 2020; Ziai et al., 2021). However, this association was not found in a cohort of 25 patients, which might be explained by a smaller sample (Li et al., 2012). Several studies also reported that CCL2 level was associated with ICH prognosis. In a cohort of 85 patients, higher serum CCL2 levels within 24 h were independently associated with functional outcomes at 7 days after ICH (Hammond et al., 2014). Another prospective study including 115 patients with ICH suggested that elevated serum CCL2 level at 6 h was associated with a 90-day worse modified Rankin Scale (mRS) score (Landreneau et al., 2018). Nevertheless, no correlation was found between CSF CCL2 level and in-hospital mortality.

It is widely acknowledged that CCL2 acts as a critical molecule in the pathogenesis of ICH, especially in the early stage. Serum CCL2 level is an effective biomarker for identifying ICH severity and predicting prognosis. Thus, CCL2/CCR2 axis is a promising target to alleviate brain injury. However, the time window for inhibiting this axis needs to be further clarified to prevent delaying the protective inflammatory responses. In addition, the prognostic value of the CCL2 level should be verified in a large-sample cohort study.

#### 3.1.2. CCL3

CCL3 is also called macrophage inflammatory protein-1α (MIP-1α), belonging to the MIP-1 CC chemokine subfamily that contains four proteins called CCL3 (MIP-1α), CCL4 (MIP-1β), CCL9/10 (MIP-1δ), and CCL15 (MIP-1γ; Maurer and von Stebut, 2004). The human CCL3 gene is identified on chromosome 17 (LD78α, LD78β, and LD78γ). It can be secreted by various mature hematopoietic cells, such as monocytes, macrophage cell lines, mast cells, Langerhans cells, fibroblasts, and lymphocytes (Cook, 1996). MIP-1α proteins could bind with CCR1, CCR3, and CCR5 to exert their chemotactic and proinflammatory effects. Moreover, it can promote homeostasis (Maurer and von Stebut, 2004). CCL3 contributes to the migration of monocytes, B lymphocytes, activated CD8^+^ T cells, NK cells, and eosinophils (Cook, 1996). It also stimulates the expression of cell adhesion molecule 1 (ICAM-1) and the production of TNF-α, IL-1, and IL-6. IL-1β, lipopolysaccharide (LPS), and HIV-1 infection induce an increased expression of CCL3 (Menten et al., 2002). In the CNS, CCL3 is released from microglia, astrocytes, hippocampal neurons, and cerebral endothelial cells (Rezaie et al., 2002; Xu et al., 2009; Chui and Dorovini-Zis, 2010; Cudaback et al., 2015). It is implicated in numerous diseases, including ischemic stroke, seizure, and traumatic injury (Arisi et al., 2015; Huang et al., 2018; Ciechanowska et al., 2020).

The roles of CCL3 in hemorrhagic stroke have not been studied as extensively as CCL2. The studies of CCL3 in ICH are relatively limited. In the mice brain after ICH, mRNA expression of CCL3 exhibited a peak increase earlier at 6 h, a decrease at 12 h, and a stable status at 24 h (Matsushita et al., 2014). Subarachnoid hemorrhage (SAH) also caused an elevated level of CCL3 (Cobelens et al., 2010). In addition, RNA sequencing analysis revealed the recruitment of macrophages *via* CCL3 in the progression of intracranial aneurysms (Aoki et al., 2019). In the preterm infants with post-hemorrhagic hydrocephalus (PHH), there was an increased level of CSF CCL3, but the team did not observe a correlation with CSF inflammatory cell counts (Habiyaremye et al., 2017). No clinical studies were searched about CCL3 in patients with hemorrhagic strokes. Therefore, the underlying mechanism of CCL3 in ICH currently needs to be clarified, requiring more directly related research.

#### 3.1.3. CCL5

CCL5, or RANTES (regulated on activation, normal T-cell expressed and secreted), was first discovered in normal T cells, acting as a critical proinflammatory chemokine (Schall et al., 1988). The production of CCL5 was also generated predominantly in CD8^+^ T cells, epithelial cells, fibroblasts, platelets, and macrophages. It has been indicated to contribute to the migration and recruitment of T cells, monocytes, dendritic cells, eosinophils, NK cells, mast cells, and basophils (Appay and Rowland-Jones, 2001). In the CNS, CCL5 was found expressed in oligodendrocytes, astrocytes, microglia, and some dopaminergic neurons (Lanfranco et al., 2017). CCL5 and its source cells participate in numerous biological processes, such as controlling pathogens, enhancing inflammation, and repairing wounds in many diseases (Levy, 2009; Marques et al., 2013).

CCL5 can attach to CCR1, CCR3, CCR4, and CCR5. There is a greater affinity for CCL5 binding to CCR1 and CCR5, but less affinity for CCR3 and CCR4 (Appay and Rowland-Jones, 2001; Blanpain et al., 2001). The CCR1 is widely expressed in multiple leukocyte types. It initiates and exacerbates inflammation and thus is considered a potential therapeutic target for autoimmune and inflammatory diseases. CCR1 signaling pathway contributes to tissue damage and inflammation *via* activating T cells, regulating Th1/Th2 cytokine polarization, and stimulating macrophage function and proteases (Cheng and Jack, 2008). CCR5 is also expressed on plenty of leukocytes, for instance, resting memory/effector T-lymphocytes, monocytes, macrophages, and immature dendritic cells (Blanpain et al., 2002). More importantly, it also serves as the main coreceptor for the entry of R5 strains of the human immunodeficiency virus (HIV-1, HIV-2). Maraviroc, an effective CCR5 antagonist at inhibiting HIV-1 entry into cells, has been approved by the United States Food and Drug Administration for the therapy of R5-tropic HIV-1 infection (Woollard and Kanmogne, 2015). Recently, accumulating evidence implicates the roles of the CCR5 axis in other infectious illnesses, autoimmune diseases, cerebrovascular events, and neurocognitive disorders (Martin-Blondel et al., 2016).

In the stroke subtype of ICH, CCL5 related signaling pathway participates in the secondary injury and provides a promising therapeutic approach for definitively managing conditions. Yan and his colleagues systematically explored the roles of CCL5 and its receptors in experimental ICH. In the hemorrhagic models induced by autologous blood, there was a notable elevation of CCL5 level starting at early 3 h, peaking at 24 h, and then decreasing at 72 h (Yan et al., 2022). The time courses of CCR1 and CCR5 expressions were parallel to that of CCL5 (Yan et al., 2021, 2022). In the hemorrhagic brain, CCR1 was localized in microglia, neurons, and astrocytes in the perihematomal area. The administration of CCR1 inhibitor with Met-RANTES significantly improved neurological deficits and decreased brain swelling after ICH *via* inhibiting inflammatory responses. The neuroprotective effects were achieved through the CCR1/TPR1/ERK1/2 signaling pathway (Yan et al., 2020). Besides, the latest research reported that Met-RANTES could preserve BBB integrity by inhibiting the CCR1/SRC/Rac1 pathway, which also partly explained the neuroprotective roles of Met-RANTES (Yan et al., 2022). The inhibition of CCR5 by Maraviroc also alleviated post-ICH neurological deficits, partially ameliorating neuronal proptosis through the CCR5/PKA/CREB/NLRP1 signaling (Yan et al., 2021). Given the effects of CCL5 and its receptors on neuroinflammation after ICH, the axis may serve as a potential target for secondary injury.

There are limited clinical data about CCL5 and its receptors levels in patients with ICH. Li collected serum samples to obtain the level of CCL5 at days 1–3, day 7, day 14, and day 30 of ICH, finding no correlation between CCL5 level and ICH severity or functional outcomes (Li et al., 2012). The results may be limited to fewer patients and later time points. In patients with subarachnoid hemorrhage, serum CCL5 level on day 7 was independently associated with clinical outcomes at discharge (Chaudhry et al., 2020). Moreover, a higher level of serum CCL5 is also related to an increased risk of DCI (Ahn et al., 2019).

In summary, further research is needed to identify the association between CCL5 level and ICH prognosis. In recent years, CCR5 has been developed as a promising therapeutic target for post-stroke recovery, including cognitive function (Joy et al., 2019; Feng et al., 2022). Therapeutic approaches targeting CCL5 and its receptors might be beneficial in ICH, especially for neurorehabilitation. However, there is no human research to discuss this correlation in ICH. CCR5 antagonist Maraviroc has been identified with promising potential for neurological recovery in ischemic stroke, and the translational process may be easier for its validated safety and wide application in AIDS patients. Therefore, the administration of Maraviroc may provide a promising therapeutic approach to managing patients with ICH.

#### 3.1.4. CCL12

CCL12, also named monocyte chemoattractant protein (MCP)-5, is a potent chemoattractant for peripheral monocytes. CCL12 is weakly active on eosinophils, whereas it is inactive on neutrophils (Jia et al., 1996; Sarafi et al., 1997). Its expression was detected in mice’s lymph nodes, macrophages, and lungs (Sarafi et al., 1997). CCR2 is the sole receptor of CCL12. In CNS diseases, CCL12 is associated with acute brain injury, including complement pathways and hypoxia-inducible inflammation (Mojsilovic-Petrovic et al., 2007; Popiolek-Barczyk et al., 2020). A recent study reported the effect of CCL12 on aged mice with ICH (Huang et al., 2020). Brain and plasma CCL12 levels increased significantly after ICH, aggravating secondary injury *via* recruiting macrophage and T cells. The genetic knockout of CCL12 could alleviate brain damage, including neurological deficits, survival rates, brain edema, and inflammatory responses. The results also provide a potential approach for ICH management, especially in the elderly with ICH.

#### 3.1.5. CCL17

CCL17 is originally named thymus-and activation-regulated chemokine (TARC) for its constitutive expression in the thymus (Yoshie and Matsushima, 2015; Catherine and Roufosse, 2021). The gene for TARC/CCL17 was mapped to chromosome 16q13 (Nomiyama et al., 1998). Murine studies have shown that steady-state TARC/CCL17 synthesis occurs in various tissues, including the thymus, lymph nodes (LNs), gut, and bronchi, but not in the spleen (Alferink et al., 2003). The cellular sources of this chemokine were Langerhans cells (LCs) and mature myeloid dendritic cells (DCs; Alferink et al., 2003). It is the first CC chemokine chemotactic for lymphoid cells but not for monocytes. CCL17 is an inflammatory chemokine with a high organ-restricted and DC-restricted expression profile (Yoshie and Matsushima, 2015). In humans, monocyte-derived DCs were shown to synthesize CCL17 in response to IL-3 and TNF-α *in vitro* cultures (Imai et al., 1999). CCL17 selectively binds to CCR4, which was found to be expressed on a fraction of Treg cells, cutaneous lymphocyte antigen (CLA), skin-homing T cells, and Th2-polarized cells (Yoshie and Matsushima, 2015). The critical role of the CCL17/CCR4 axis in immune suppression by Treg cells has also been well documented in animal models and human samples. CCL17 is expressed by DCs in the autoimmune encephalomyelitis models and promotes the pathogenesis of the disease (Ruland et al., 2017). In the hippocampus, CCL17 was identified as a homeostatic neuromodulator affecting the presence and morphology of microglia and synaptic transmission (Fülle et al., 2018).

Regarding the role of CCL17 in ICH, its expression increased after ICH from 6 h, reached a peak on day 5, and then decreased on day 7, while CCR4 significantly increased from 12 h to 5 days (Deng et al., 2021). The recombinant CCL17 (rCCL17) administration might promote hematoma resolution by increasing the expression of CD163 on microglia/macrophages, further reducing perihematomal edema and improving neurobehavior outcomes. The haptoglobin-CD163 scavenging system plays a critical role in the endogenous elimination of blood metabolites from the ICH-affected brain (Garton et al., 2017). In addition to the effects of CCL17 on hematoma resolution, the axis also could alleviate neuroinflammation and neuronal apoptosis *via* the CCR4/PI3K/AKT/Foxo1 signaling pathway at 72 h post-ICH (Deng et al., 2021). In experimental SAH rats, CCL17 presents neuroprotective effects by activating CCR4/mTORC2 axis in microglia (Zhang A. et al., 2022). There is no other clinical study about CCL17 in patients with ICH. Given the neuroprotective roles of CCL17, its activation has become a promising therapeutic approach for early therapy of ICH and contributing to hematoma absorption. Future study needs to focus more on the crossing influence of CCL17 and Treg cells.

### 3.2. CXC chemokine family

Based on the presence or the absence of a tripeptide motif glutamic acid–leucine–arginine (ELR) in the N-terminal domain, CXC chemokines are stratified into ELR^+^ or ELR^−^ molecules. The CXC-ELR^+^ chemokines are chemoattractants mainly for neutrophils, compared to CXC-ELR-chemokines primarily attracting lymphocytes and monocytes (Bajetto et al., 2001). The presence of ELR has also been proposed to induce angiogenesis and to be chemotactic for endothelial cells (Bizzarri et al., 2006), including CXCL1, CXCL2, CXCL3, CXCL5, CXCL6, CXCL7, and CXCL8 (Murphy et al., 2000). In contrast, the non-ELR-CXC chemokines guide the recruitment of activated T cells and possess anti-angiogenic properties, such as CXCL4, CXCL10, and CXCL9 (Erdem et al., 2007; Repnik et al., 2015). Additionally, CXCL12 was observed to induce neovascularization *in vivo* (Dimova et al., 2019).

The family also plays an essential role in the pathogenic events after ICH. CXCL1 and CXCL2 were significantly elevated in the ipsilateral hemisphere at 6 h after induction of ICH (Matsumoto et al., 2020). CXCL2 exerts key effects in ICH models with axonal tract injury (Katsuki and Hijioka, 2017). Here, we mainly introduce CXCL8 and CXCL12.

#### 3.2.1. CXCL8

CXCL8, also known as interleukin 8 (IL-8), is the first identified chemokine (Yoshimura, 2015). IL-8 is released by multiple cell types, such as monocytes, lymphocytes, granulocytes, fibroblasts, endothelial cells, and epithelial cells (Zlotnik et al., 1999). IL-1 and TNF-α can activate IL-8 gene expression through the IL-1R and TNFR signaling pathways (Hoffmann et al., 2002). CXCL8 is initially thought to act as a neutrophil mobilizer and engaged in acute inflammation, but it is also discovered to have chemotaxis to endothelial cells contributing to angiogenesis (Matsushima et al., 2022). CXCL8 interacts with CXCR1 and CXCR2 receptors expressed on T cells, monocytes, NK cells, basophils, and other non-hematopoietic cells in regulating angiogenesis, pain, and cardiovascular diseases (Strieter et al., 1995; Liu et al., 2016; Matsushima et al., 2022). In the CNS, the sources of CXCL8 include active microglia, astrocyte, endothelial cells, and infiltrated neutrophils, which is upregulated in pathological conditions (Lu et al., 2005; Vallès et al., 2006). CXCR2 has been reported to express on activated microglia or astrocytes surface. The CXCL8 and its receptors seem to be involved in various brain pathologies, such as ischemic brain injury, multiple sclerosis, and traumatic brain injury (TBI; Semple et al., 2010).

In the pathology of ICH, the CXCL8 gene was expressed upregulated in the perihematomal areas obtained from deceased patients (Rosell et al., 2011). CSF level of CXCL8 is correlated with relative PHE volume in patients with ICH, which supports CXCL8 as a biomarker for ICH severity (Ziai et al., 2021). Heme oxygenase-1 (HO-1) inhibitor was also reported to regulate the expression of CXCL8 (Fan and Mu, 2017). CXCL8 may be a novel candidate susceptibility gene for ICH. Notably, it may serve as a predictive marker for ICH severity, especially for serum concentrations.

#### 3.2.2. CXCL12

CXCL12 (also named stromal cell-derived factor-1, SDF-1), interacting with CXCR4 and CXCR7, is one number of homeostatic chemokines. Thus, it is produced by bone marrow stromal cells and epithelial cells in many other organs, which is indispensable for lymphopoiesis and embryogenesis (Nagasawa et al., 1996; Janssens et al., 2018b). CXCL12 is also a crucial molecule in the procedure of inflammation, mediating the activation and migration of monocyte, macrophage, Treg cell, and microglia (Li Y. et al., 2019; Wang et al., 2019; Mai et al., 2021; Zhang L. et al., 2022). CXCL12 mainly recruits progenitor cells and white blood cells through CXCR4, while CXCR7 mainly inhibits the CXCL12/CXCR4 axis (Janssens et al., 2018a). Its secretion increases in these organs during tissue damage such as heart infarction, cerebral ischemia, toxic liver damage, excessive bleeding, and total body irradiation. Furthermore, CXCR4 also functions as a co-receptor for virus entry into T cells (Huang et al., 2021). It is now recognized that CXCL12/CXCR4 signaling regulates the development of nervous tissue in different ways, particularly due to its effects on cell migration and axon guidance (Mithal et al., 2012).

The related research about the effects of CXCL12/CXCR4 on ICH is rare and controversial. A cohort study was conducted on 105 ICH patients, indicating that baseline serum CXCL12 concentrations were enhanced after ICH. Higher concentrations of CXCL12 were related to ICH severity and poor outcomes (Shen et al., 2017). Yu suggested the neuroprotective and anti-inflammatory action of CXCR4 antagonist CX807 (Yu et al., 2020). The results may be explained by inhibiting inflammatory and apoptotic markers, such as TLR4, TNF-α, IL-6, and CD8. In addition, CXCL12 has been reported to stimulate endothelial progenitor cells (EPCs) to induce angiogenesis through the CXCR4 pathway after ICH (Li et al., 2015). However, human data suggested that patients harboring the Tp53 Arg72 Pro single-nucleotide polymorphism (SNP) had better functional outcomes but higher SDF-1α levels (Rodríguez et al., 2017). In TBI models, pharmacological blockers of CXCR4 improved recovery (Friedman-Levi et al., 2021). These studies provide a new therapeutic potential for preventing and reducing ICH-related injury, but it needs to clarify the underlying mechanisms of a CXCR4 antagonist in ICH.

### 3.3. CX3C chemokine family

CX3CL1 (Fractalkine, FKN) is the only member of the CX3C chemokine family as a transmembrane protein or soluble chemokine. It is constitutively expressed in neurons, microglia, astrocytes, and vascular endothelial cells (Harrison et al., 1998; Yoshida et al., 2001). CX3CR1 is the exclusive receptor of CX3CL1 existing on the surface of neuron and microglial cells (Harrison et al., 2001; Hatori et al., 2002). The CX3CL1/CX3CR1 axis in physiological conditions appears critical for normal brain functions (Zhan et al., 2014). After brain injury, CX3CL1 binding to CX3CR1 regulates the activation of microglial cells and mediates interactions between neurons and microglia (Chapman et al., 2000). Nevertheless, current data are still somewhat controversial. Many studies have suggested the neuroprotective effects of CXC3R1 in ischemic stroke and other diseases (Meucci et al., 2000; Wang et al., 2018).

In recent decades, substantial data from ICH have consistently demonstrated that CX3CL1/CX3CR1 signaling can achieve some neuroprotective effects in the pathology of ICH, including contributing to the absorption of hematoma size and reducing cellular death. Gaetani first highlighted the expression of CX3CL1 and CX3CR1 in the human brain after ICH and TBI, finding that significant upregulation of CXC3L1/CXC3R1 might be involved in limiting brain damage (Gaetani et al., 2013). You further speculated on the specific mechanism of CX3CL1 promoting hematoma clearance (You et al., 2022). The expressions of both CX3CL1 and CX3CR1 increased early at 6 h of ICH onset, peaked at 3 days, and then decreased gradually in the following days. By administrating CX3CL1, it could increase the chemotactic ability of microglia toward the hematoma, accelerate hematoma absorption, and thus improve neurological function recovery. Moreover, PPAR-γ was found to mediate the increase in the CD163/HO-1 axis expression and erythrophagocytosis induced by CX3CL1 in microglia. However, Min (Min et al., 2016) have suggested that the CX3CR1^+^ cells after ICH are mainly due to macrophage infiltration rather than microglia proliferation, which increased from 1 day until 7 days of ICH. Numerous macrophages were polarized to the M2 phenotype at delayed time points (3 and 7 days), playing a protective role by presumably facilitating recovery from ICH injury. The study is partially inconsistent with previous studies and may be explained by the discrepancy between the animal model and different stages of ICH. Furthermore, stem cell therapy has emerged as a promising therapeutic strategy for ICH, but it has low retention and engraftment after delivery (Gao et al., 2018).

Interestingly, a study reported that the overexpression of CX3CR1 in adipose-derived stem cells promotes cell migration and functional recovery after experimental ICH, which might contribute to the development of stem cell therapy (Li G. et al., 2019). Clinically, a prospective cohort including 30 ICH patients recently showed the relevance of serum CX3CL1 concentration and better prognosis (You et al., 2022). Given the effects of CX3CL1 on hematoma absorption, it might be a promising target for ICH treatment in the ultra-early stage. Because of inadequate data, more research is required to explore its neuroprotective effects further.

## 4. Chemokines and their receptors as drug targets

Based on the up-regulated expression of chemokines and their receptors after ICH, some studies primarily target chemokine receptors with pharmacological drugs in ICH models, enabling the system as a preferential approach. We summarized the usage and effects of these drugs in preventing ICH-induced injury, including Propagermanium, Maraviroc, and Reparixin (Table 4). Nevertheless, clinical ICH trials about drugs targeting chemokine or receptors currently lag. We also list more studies of corresponding drugs in clinical usage or under clinical trials in other cardiovascular and cerebrovascular diseases (Table 5). For example, the CCR5 inhibitor Maraviroc is the first targeting drug tested in patients with IS (Francisci et al., 2019). Relevant results are not well-documented, but it opens a window for neurorehabilitation after IS (Feng et al., 2022). In addition, it was reported that Maraviroc could attenuate atherosclerotic progression in HIV patients, as well as anti-CCR2 monoclonal antibody MLN1202 for its anti-inflammation (Gilbert et al., 2011; Francisci et al., 2019). JVS-100 was developed to treat ischemic cardiovascular diseases as a nonviral, naked DNA plasmid encoding human CXCL12. The endomyocardial injection of JVS-100 was safe and improved heart failure symptoms *via* the critical role of the CXCL12/CXCR4 axis in tissue repair (Penn et al., 2013).

**Table 4:** Summary of studies evaluating the effects of drugs targeting chemokines and their receptors in ICH.

Drug	Mechanism	Disease model	Drug administration details	Main effects	Signaling pathway	Reference
Propagermanium	CCR2 inhibitor	collagenase	Oral gavage: 3 times a day (25 mg/kg/day) for 1 day or 3 days	Reduce brain edema, improve neurobehavioral functions	CCL2-CCR2-p38 MAPK	Guo et al. (2020)
Maraviroc	CCR5 inhibitor	autologous blood	Intranasal delivery: once per day (50 μg/kg or 150 μg/kg or 450 μg/kg) for 3 days	Improve neurobehavioral deficits, decrease neuronal pyroptosis	CCR5/PKA/CREB	Yan et al. (2021)
Reparixin	CXCR1/2 antagonist	collagenase	Intravenous and intraperitoneal administrations: three times a day (15 mg/kg) for 3 days	Ameliorate neurological dysfunction	/	Matsushita et al. (2014)

**Table 5:** Summary of drugs in clinical usage or under clinical trials targeting on chemokines and chemokine receptors in cerebrovascular diseases.

Target	Drug	Conditions	Aim of study	Study outcomes and status	Phase	Clinical trial number
CCL2	Bindarit	Coronary restenosis	Assess the efficacy and safety of different dosages in preventing restenosis	Completed. No significant differences in in-segment, and MACE rates; significant reduction in the in-stent late loss	Phase 2	NCT01269242 Colombo et al. (2016)
CCR2	MLN1202	Atherosclerosis	Evaluate the potential of MLN1202 to reduce circulating levels of C-reactive protein in patients with risk factors for atherosclerotic cardiovascular disease	Completed. MLN1202 treatment resulted in significant reductions in high-sensitivity C-reactive protein levels	Phase 2	NCT00715169 Gilbert et al. (2011)
CCR5	Maraviroc	Stroke	Maraviroc to augment rehabilitation outcomes after stroke (MAROS)	Terminated. Only 2 participants were enrolled; No data displayed; Participants exited study prior to 6-month assessment	Phase 2/3	NCT03172026
CCR5	Maraviroc	Stroke	RCT to analyze the effect of Maraviroc and exercise to improve extremity recovery after a stroke	Not yet recruiting	Phase 2	NCT04789616
CCR5	Maraviroc	Post-stroke cognitive impairment	Investigate the safety and efficacy of Maraviroc in post-stroke impairment	Recruiting. No results.	Phase 2	NCT04966429 Assayag et al. (2022)
CCR5	Maraviroc	Atherosclerosis in HIV Patients	Efficacy of Maraviroc in modulating atherosclerosis in HIV patients	Completed. Maraviroc intensification modulates atherosclerotic progression in HIV-suppressed patients at high cardiovascular risk	Phase 4	NCT03402815 Francisci et al. (2019)
CXCL12	JVS-100	Severe peripheral arterial disease	Investigate the efficacy of JVS-100 on composite endpoints of wound progression, healing, and limb loss in patients with severe peripheral arterial disease	Unknown. JVS-100 failed to improve wound healing or hemodynamic measures at 3 months	Phase 2	NCT02544204 Shishehbor et al. (2019)
CXCL12	JVS-100	Ischemic heart failure	Assess the safety and efficacy of using JVS-100 to treat heart failure	Completed. JVS-100 improved heart failure symptoms in patients with ischemic cardiomyopathy	Phase 1	NCT01643590 Penn et al. (2013)
CXCL12	ACRX-100	Ischemic heart failure	Evaluate the safety of a single escalating dose of ACRX-100 in adults with ischemic heart failure	Completed. No results posted	Phase 1	NCT01082094
CXCR2	AZD5069	Atherosclerotic coronary disease	Determine whether CXCR2 inhibition will improve coronary endothelial function in patients following PCI for atherosclerotic coronary disease	Ongoing. No results posted	Phase 2	EudraCT 2016-000775-24
CXCR4	POL6326	Large reperfused ST-elevation myocardial infarction	Investigate the effects of POL6326 as a stem cell mobilizing agent on cardiac function and infarct size	Completed. No results posted	Phase 2	NCT01905475

MACE, major adverse cardiovascular events; RCT, randomized controlled trial; HIV, human immunodeficiency virus; PCI, percutaneous transluminal coronary intervention.

Chemokines and their receptors have been consistently identified as critical molecules of the pharmacological target. Its characteristic of regulating immune cell migration and recruitment contributes to the progression of ICH, which prompts the research from bench to bedside. On the one hand, chemokines can be developed as effective biomarkers for predicting ICH severity and prognosis, as mentioned above, such as CCL2, CXCL12, and CX3CL1. On the other hand, modulation of chemokine expression can be further explored to alleviate secondary injury despite the low clinical translation rate. For example, Maraviroc also has a promising potential in facilitating the translation of basic science to the clinical setting in ICH for its confirmed safety and neuroprotective effects on ICH. The role of Maraviroc in post-stroke recovery may also be the main impetus for rehabilitation after ICH. One major drawback in limiting its translational potential is the redundancy in the function through both spatial and temporal differential expression. Thus, chemokine receptor antagonists may be a more efficient approach with specificity for multiple targets. Moreover, several mechanisms are involved in ICH pathophysiology more than neuroinflammation. Thus, therapies of chemokines should be combined with other targets to test the effects on ICH.

## 5. Conclusion

The mechanisms underlying ICH-induced brain injury are currently unclear. Because chemokines are expressed temporally and spatially in the pathogenesis of acute ICH and post-ICH recovery. It will be vital to determine when and how chemokines influence immune responses. These mediators have various functions, including pro-inflammatory, anti-inflammatory, homeostatic, angiogenic, and neuromodulatory effects, which are intertwined and relatively complex. Many cell types produce chemokines in damaged brain tissues after ICH, including resident cells and infiltrating immune cells. Clarifying which cell types are the primary sources of chemokines in human or animal ICH is challenging. In addition, the interactions between chemokines and their receptors are highly relevant to the clinical procedure. Correspondingly, different chemokine receptors are also expressed on many cell subtypes in the brain and from peripheral immune systems. However, it is exactly because of these multiple functions serving a promising therapy with multi-targets on several chemokines or different mechanisms.

In summary, the local concentrations of chemokines in CSF or serum may provide promising predictors for ICH severity to aid decision-making in managing conditions. Their interactions with receptors determine the progression and outcome of relevant brain damage, contributing to developing new targets for hematoma absorbance, alleviating brain edema, and neurological recovery. Recently, rapid technology advances will shed light on the specific sources and activating time windows of different chemokines in ICH. Furthermore, more signaling pathways and transcription factors will be illustrated. It is conceivable that the work targeting specific chemokines and/or their receptors can effectively eliminate injury and improve prognosis in treating ICH. Nevertheless, exploring more new insights and discoveries on chemokines and their interactions with receptors is warranted. Moreover, more studies are necessary before those new findings can be translated into clinical targets.

## Author contributions

JW: conceptualization, data curation, investigation, methodology, project administration, visualization, and writing the original draft. LB: conceptualization, data curation, investigation, methodology, project administration, and visualization. YD, DW, and RJ: conceptualization and methodology. JL: supervision and writing-review and editing. XZ: funding acquisition, resources, supervision, and writing-review and editing. All authors contributed to the article and approved the submitted version.

## Funding

The research was supported by the National Key Research and Development Program of the People’s Republic of China (grant nos. 2018YFC1312200/2018YFC131224 and 2018YFC1705003), CAMS Innovation Fund for Medical Sciences (2019-I2M-5-029), Beijing Hospitals Authority Innovation Studio of Young Staff Funding Support (202112), and Beijing Municipal Committee of Science and Technology (Z201100005620010).

## Conflict of interest

The authors declare that the research was conducted in the absence of any commercial or financial relationships that could be construed as a potential conflict of interest.

## Publisher’s note

All claims expressed in this article are solely those of the authors and do not necessarily represent those of their affiliated organizations, or those of the publisher, the editors and the reviewers. Any product that may be evaluated in this article, or claim that may be made by its manufacturer, is not guaranteed or endorsed by the publisher.
